# Treating Aggression and Self-destructive Behaviors by Stimulating the Nucleus Accumbens: A Case Series

**DOI:** 10.3389/fneur.2021.706166

**Published:** 2021-10-11

**Authors:** Marek Harat, Michał Kiec, Marcin Rudaś, Marcin Birski, Jacek Furtak

**Affiliations:** ^1^Department of Neurosurgery and Neurology, Collegium Medicum of the Nicolaus Copernicus University, Toruń, Poland; ^2^Neurosurgery Clinic, 10th Military Research Hospital, Bydgoszcz, Poland; ^3^Clinic of Neurosurgery and Neurology, The Department of Neurosurgery and Neurotraumatology with the Treatment Improvement Sub-unit, Jan Biziel University Hospital No. 2, Bydgoszcz, Poland

**Keywords:** deep brain stimulation, nucleus accumbens, aggression, self-destructive, behavior

## Abstract

Self-destructive and aggressive behaviors can have a significant impact on the quality of life of affected individuals and their carrers. While deep brain stimulation (DBS) has been applied to the treatment of self-destructive and aggressive behaviors in isolated cases, clinical data on this treatment modality are still lacking. We therefore assessed responses to treatment with bilateral DBS of the nucleus accumbens in six patients with severe self-destructive and aggressive behaviors. Three patients had Tourette syndrome and three had other underlying predispositions including obsessive compulsive disorder, cerebral palsy, encephalitis, and epilepsy. Patients were followed up for between 2 and 7 years, and patients were assessed using the Modified Overt Aggression Scale (six patients) and the Buss-Perry Aggression Questionnaire (three patients able to complete the questionnaire on their own). DBS reduced self-destructive and aggressive behaviors by 30–100% and by an average of 74.5%. Patients with Tourette syndrome responded better to DBS and improved by 27.3% according to the Buss-Perry Aggression Questionnaire. These results suggest that nuclei accumbens stimulation may be an effective treatment for aggressive and self-destructive behaviors regardless of etiology.

## Introduction

Self-destructive and aggressive behaviors can reduce the quality of life of patients and their carers and represent a serious management problem. In their most severe forms, these behaviors can threaten the patient's life due to serious self-mutilation. They also cause social disability because such patients are often removed from schools and care institutions. Patients with the most severe conditions live with direct coercive mechanical restraint (e.g., belts, strap) 24 h a day, often for many years. Intractable aggression is not a pathological entity defined as a diagnosis in the Diagnostic and Statistical. Manual of Mental Disorders IV; instead, aggression is a behavior found in a variety of psychological disorders including cognitive disorders, substance abuse, psychosis, and personality disorders (1). These conditions can be extremely difficult to treat conservatively, and the most effective therapeutic strategies are based on behavioral psychotherapy techniques.

There have been at least 50 years of attempts to surgically treat aggressive and self-destructive behaviors. First attempts included the destruction of the basilar and medial-cortical amygdala in patients with epilepsy and aggressive behavior (1–8). In the seventies, Sano et al. reported good results with posterior medial hypothalamus ablation (9). The introduction of deep brain stimulation (DBS) increased the safety and efficacy of functional neurosurgery, resulting in several attempts to apply DBS to the posterior medial hypothalamus (10, 11), the basolateral part of the amygdala (8, 12), and the nucleus accumbens (11, 13) in patients with severe self-destructive and aggressive behaviors.

**Figure 1:**
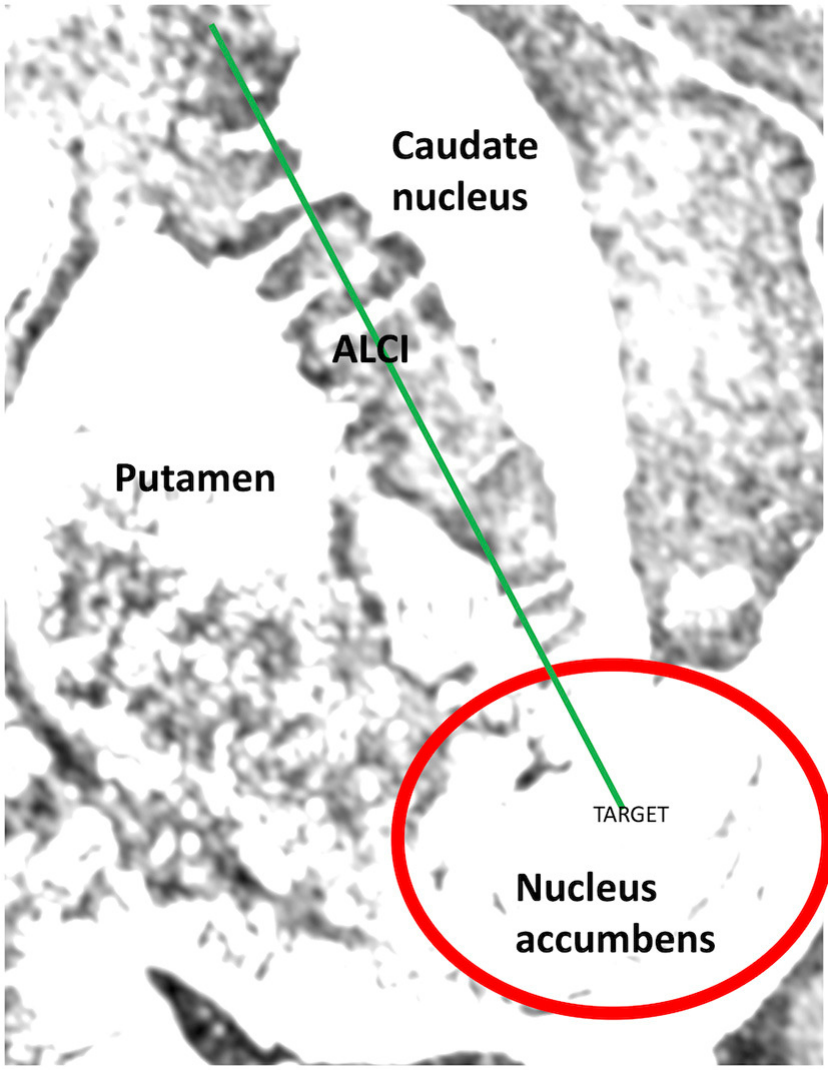
Nucleus accumbens and the site of neurostimulation (red circle).

**Figure 2:**
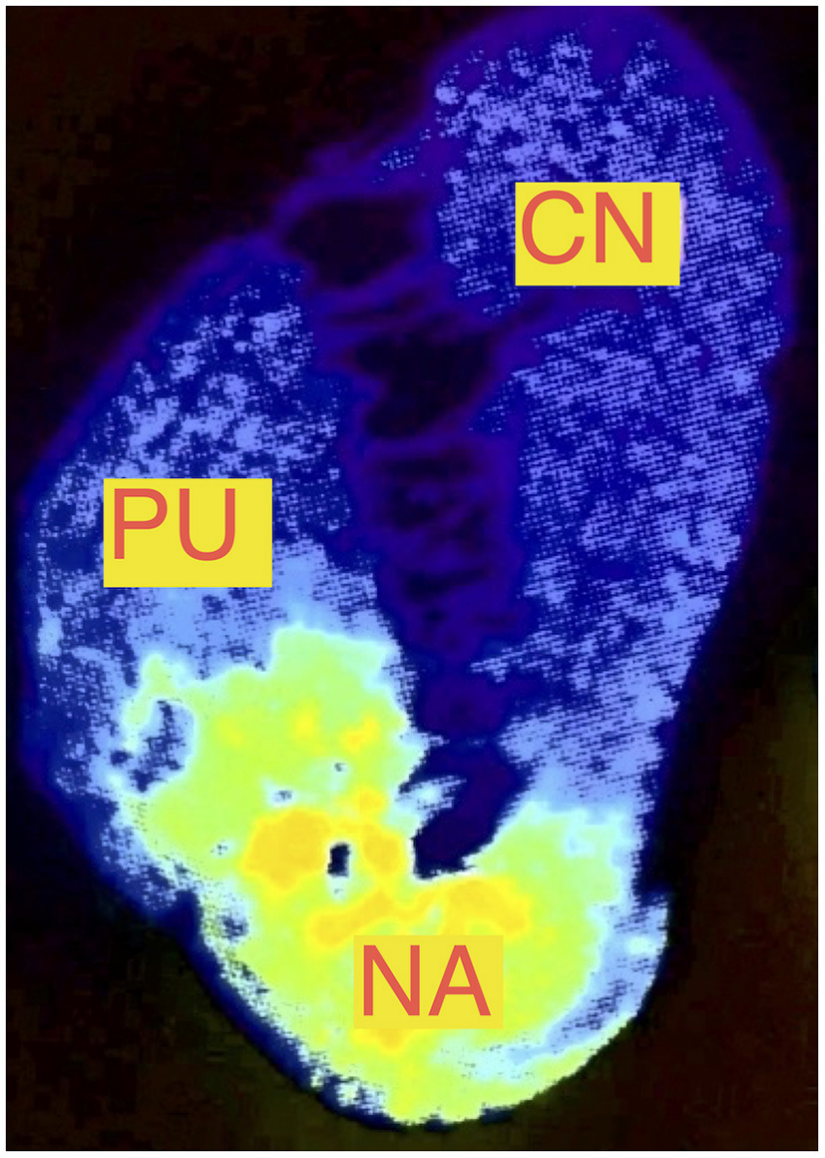
PET examination of the nucleus accumbens showing the distribution of dopaminergic D3 receptors (NA, nucleus accumbens; CN, caudate nucleus; Pu, putamen) based on Gurevich et al. (16).

The nucleus accumbens is located in the ventral part of the striatum and has two functionally important parts: the medially located core and the shell, which surrounds the core on the medial, lateral, and ventral sides. Below the anterior limb of internal capsule, the nucleus accumbens, putamen, and caudate nucleus are joined and their boundaries are difficult to precisely define; this area was chosen for stimulation in this case series (as shown in Figure 1). The diversification of the nucleus accumbens into the core and shell was traditionally of interest to basic neuroscientists studying emotional processes and adaptive behavior, but more generally the nucleus accumbens has been of interest due to its importance in the pathogenesis of neuropsychiatric disorders (14). The nucleus accumbens appears to act as an emotional-motoric switch, since it mainly receives information from the key limbic structures (the amygdala and the hippocampus), and various pharmacological agents applied to nucleus accumbens induce locomotor activation (15). As a part of ventral striatum, it is also an active area in the dopaminergic neurotransmitter system (Figure 2) (16). Many subsequent reports have described much larger projections from the ventral striatum to the medial-dorsal nucleus of the thalamus, which is more closely associated with the pre-frontal cortex than the pre-motor cortex (14). This discovery paved the way for the modern concept of the existence of parallel cortico-subcortical loops, which formed the basis for explaining the symptoms associated with mental disorders (17, 18). Functional tests such as with positron emission tomography (PET) have now indicated that the ventral striatum is activated while eating, during intercourse, and when taking drugs, drinking alcohol, and gambling, all of which can be addictive.

Here we present six cases that illustrate that the nucleus accumbens can be an effective and safe target for DBS in patients with various diseases dominated by symptoms of aggressive and self-destructive behavior.

## Case Series

Six patients (three men and three women) were included in the study. All had been ineffectively treated conservatively for aggressive and self-destructive behaviors for at least 5 years by a multidisciplinary team of psychiatrists, neurologists, neurosurgeons, and allied health professionals. DBS was approved by the bioethics commission on an individual basis (Military Medical Chamber Bioethics Commission approvals 115/13, 124/14, and 147/17). Sixteen years old patient and both of his parents signed informed consent and in two cases of adult incapacitated patients' legal representatives (their parents) signed informed consent, in all other cases patients signed informed consent themselves.

The clinical details and follow-up of the patients are summarized in Table 1. Three patients had Tourette syndrome. The nuclei accumbens was chosen as a DBS target in these patients because in two patients motor and vocal tics were not a significant clinical problem, their poor quality of life of patients instead dominated by aggressive and self-destructive behavior. The other Tourette syndrome patient had previously received bilateral stimulation of the internal globus pallidus, resulting in a significant reduction in motor and vocal tics, and the patient's main problems were aggressive and self-destructive behaviors, so nucleus accumbens stimulation was selected as second-stage treatment. One patient had cerebral palsy, obsessive compulsive disorder (OCD), personality disorders, moderate intellectual disability, and congenital deafness. She communicated with her parents using sign language and she had functioned well during the early years of her life, graduating from a special needs school and fully participating in family life. Around the age of 20, she developed aggressive and self-destructive behaviors unresponsive to conservative treatment, and after 5 years of unsuccessful conservative treatment attempts, she spent most of the day under direct restraint in her bed. Another patient was treated for OCD and behavioral disorders that had appeared in childhood, and she showed a tendency to self-mutilate her face. The final patient was the most severely affected, having been diagnosed with autism, had encephalitis as a child, and since then had required treatment for epilepsy. He had been hospitalized many times due to aggression and self-destruction disorders, without any improvement. He had spent most of his time over the last 3 years under direct restraint. The three patients without Tourette syndrome spent the last months before surgery under direct coercion due to aggressive and self-aggressive behaviors that threatened their lives.

**Table 1:** The clinical and follow-up details of the case series.

**Patient**	**Diagnosis**	**Age at time** **of surgery** **(years)**	**Time from** **symptom onset** **to surgery** **(years)**	**Duration of** **DBS treatment** **at the time** **of evaluation** **(years)**	**Symptoms**	**Medications taken** **before surgery**	**Medications taken** **during treatment**	**Medications taken** **at time of** **evaluation**	**Other treatment**	**Complications**
1	Tourette syndrome	38	16	2	Aggression and self-destructive behavior vocal tics, motor tics	No mediations taken	Escitalopram 20 mg once a day Topiramate 20 mg one a day Clonidine 75 mg twice a day Perphenazine 8 mg + 12 mg	Escitalopram 20 mg once a dayTopiramate 20 mg one a dayClonidine 150 mg twice a dayPerphenazine 12 mg + 20 mg	GPi DBS prior to NAcc DBS	Inflammatory complications, explantation of GPi DBS system
2	Tourette syndrome	16	–	3	Aggression and self-destructive behavior, motor and vocal tics, obsessive-compulsive behavior	No mediations taken	No mediations taken	No mediations taken	None	None
3	Tourette syndrome	19	–	7	Aggression and self-destructive behavior, vocal tics, motor tics, obsessive-compulsive behavior	No mediations taken	Tiapride 100mg one a day Hydroxyzine 50 mg once a day Valproic acid 1g once a day Citalopram 40 mg one a day Carbamazepine 0.4 g twice a day Aripiprazole 10 mg once a day	Tiapride 100 mg one a dayHydroxyzine 25 mg twice a dayTopiramate 50 mg twice a dayPimozide 1 mg once a day	None	IPG malfunction due to patient non-compliance
4	Unspecified behavioral and emotional disorders	22	7	5	Aggression and self-destructive behavior motor tics, movement stereotypes, intrusive activities	Quetiapine 40 mg one a dayClozapine 25mg twice a dayLorazepam 5mg twice a dayOxcarbazepine 900 mg twice a day	Clozapine 25mg twice a day Lorazepam 2.5 mg twice a day Oxcarbazepine 600 mg twice a day Olanzapine 5 mg twice a day Paroxetine 20 mg twice a day	Lorazepam 1 mg twice a dayOxcarbazepine 300 mg + 600 mgRisperidone 2 mg twice a day	Posterior thalamus DBS after Nacc DBS	Inflammatory complications, explantation of posterior thalamus DBS system
5	Unspecified behavioral and emotional disorders	25	10	6	Self-destructive behavior and aggression toward the environment, stereotypical and intrusive activities, inversion of circadian rhythms	No mediations taken	Paroxetine 20 mg twice a day	No mediations taken	Posterior thalamus DBS prior to Nacc DBS	Posterior thalamus DBS system impedance increase, system shutdown
6	Unspecified behavioral and emotional disorders	24	–	2	Self-destructive behavior, motor stereotypes and echolalia; self-aggression; obsessive thoughts and hygiene compulsions	Valproic acid 500 mgtwice a dayChlorprothixene 50 mgonce a dayAripiprazole 15 mgone a day	No mediations taken	No mediations taken	None	None

**Figure 3:**
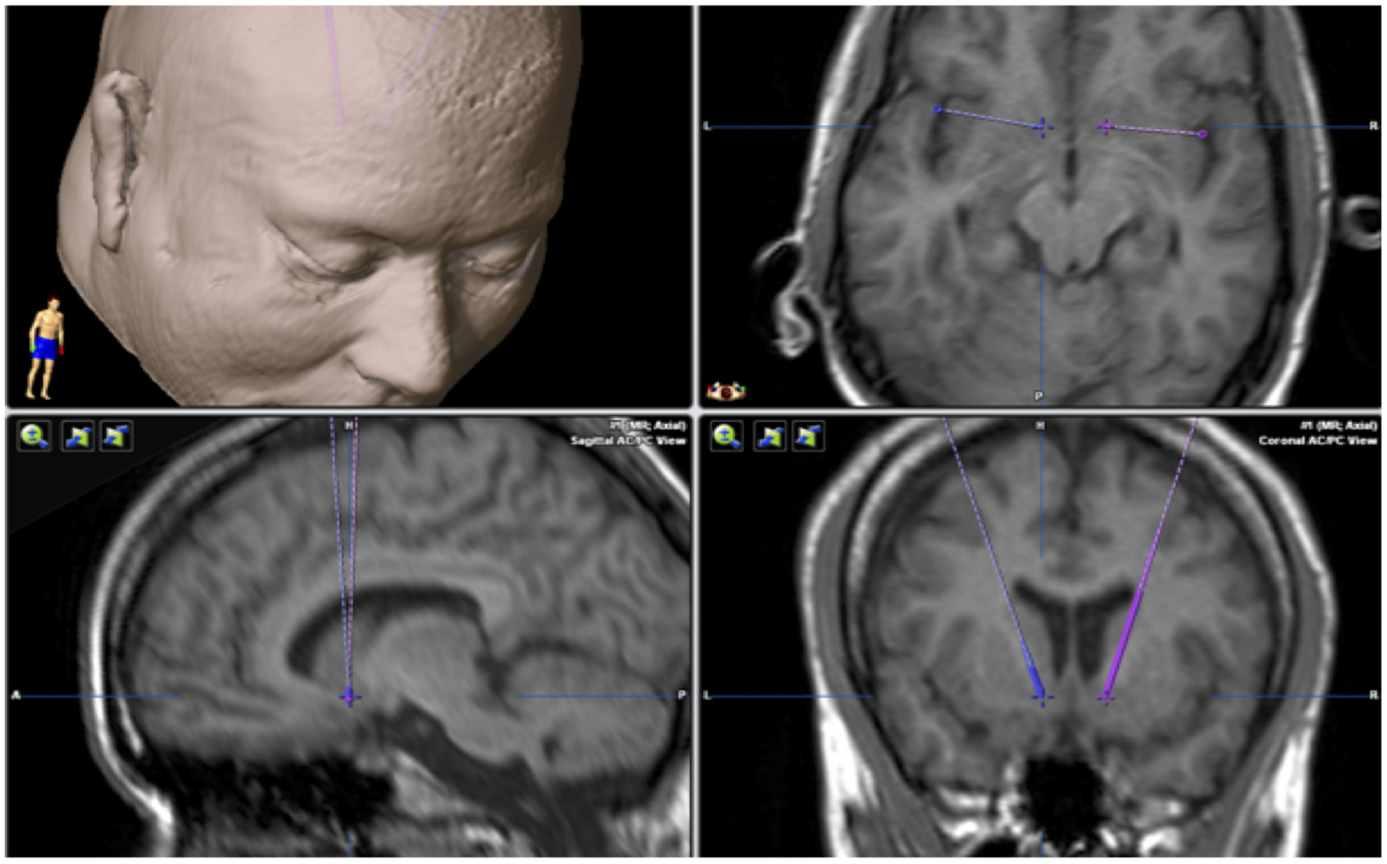
Stereotactic planning. MRI examination with a planned target in the nucleus accumbens in 3 planes. The cross points to the target, and the solid and dashed lines indicate the course of the electrodes in the brain.

Stereotactic frames were sited under general anesthesia in the operating room. CT and MRI (1.5 T) images were fused. The nucleus accumbens and the course of the electrodes were determined on MRI 1 images at times T1 and T2 in cross-sectional, sagittal, and frontal sections (Figure 3). The target was selected based on the T1 MRI image, and the electrode was implanted through the anterior limb of the internal capsule. Rather than being guided by the AC-PC coordinates, the target was usually 7 mm lateral to the intercommissural line and 4–5 mm ventral/2–3 mm anterior to the anterior commissure.

The neurosurgeon led the programming, and the individual trajectories and targets for each patient are shown in the Supplementary Materials. Electrodes were implanted in a typical way without intraoperative electrophysiological examination. After electrode implantation, intraoperative CT and pre-operative MRI fusion were performed again. After confirming that the positions of the electrodes were in accordance with the preoperative plan, the stereotactic frames were removed and the pulse generators were implanted. The stimulation parameters were adjusted in each patient to obtain a satisfactory effect. Stimulation was attempted with one or more contacts either with mono or bipolar current. When there was no clinical effect, the stimulation parameters were changed, starting with a lower current and then increasing the current gradually, always to above 5 mA. The deepest contact was used in each case. The area of stimulation is shown in Figure 1.

Pre- and post-treatment aggression were evaluated in all patients using the Modified Overt Aggression Scale (MOAS) (19). In addition, the condition of the three least affected patients with Tourette syndrome was assessed using the Buss-Perry Aggression Questionnaire (Amity version) (20), since they were able to complete the questionnaire on their own. Assessments were performed before surgery and in the postoperative period, with analyses performed between 2 and 7 years after surgery (Table 1). After implantation, each patient was under the care of a neurosurgeon who assessed the patient's condition and adjusted the stimulation parameters accordingly, with consultations taking place at different times and the patients' conditions determined on the date of consultation. However, consultations took place at least every 12 months. The condition of the patient used here was assessed during the last consultation before the completion of data collection. Patients with Tourette's syndrome were also assessed for the presence of tics; however, in this paper, we present only assessment of aggressive and self-destructive behaviors, because these were the main symptoms and the reason for treatment (Table 1).

Improvements of between 30 and 100% were observed on the MOAS, with an average improvement of 74.5% (Table 2). Patients with Tourette syndrome had better outcomes than the other three patients, with an average improvement of 95.0%. However, even the most severely affected patient with autism and epilepsy improved by 30%, which resulted in him no longer requiring direct restraint and allowing him to function as part of his family, albeit limited to the family home and usually in his room listening to music. Nevertheless, the patient's family regarded the treatment results as very good and exceeded their expectations.

**Table 2:** MOAS results.

**MOAS**	**1** **Pre**	**1** **Post** **26 months**	**2** **Pre**	**2** **Post** **39 months**	**3** **Pre**	**3** **Post** **82 months**	**4** **Pre**	**4** **Post** **56 months**	**5** **Pre**	**5** **Post** **71 months**	**6** **Pre**	**6** **Post** **28 months**
Verbal aggression	4	1	6	1	7	1	10	10	0	0	3	1
Aggression against Property	2	0	12	0	0	0	20	8	10	0	12	12
Autoaggression	9	0	18	0	0	0	30	12	18	0	18	12
Physical Aggression	8	0	24	0	4	0	40	40	0	0	4	0
Sum	23	1	60	1	11	1	100	70	28	0	37	25
**Improvement (%)**		**95.7**		**98.3**		**90.9**		**30.0**		**100.0**		**32.4**

The patient who had a complete symptom withdrawal had cerebral palsy, personality disorder, OCD, moderate intellectual disability, and congenital deafness. After surgery, the patient returned to her pre-aggressive state and was able to go on overseas holidays, bicycle trips, visit friends, and go shopping. Before surgery, she spent most of her time in direct restraint.

Patients with Tourette syndrome were also assessed with the Buss-Perry Aggression Questionnaire and showed an average improvement of 27.4% (11.8, 20.0, and 50.4%; Table 3). The best results were obtained for the assessment of physical aggression and hostility, while the smallest improvement was seen for verbal aggression.

**Table 3:** Buss-Perry Questionnaire (Amity version) results.

**Buss-Perry Questionnaire**	**1 Pre**	**1 Post** **26 months**	**2 Pre**	**2 Post** **39 months**	**3 Pre**	**3 Post** **82 months**
Physical Aggression	30	14	35	13	23	18
Verbal Aggression	12	13	15	14	24	24
Anger	31	30	29	14	26	27
Hostility	37	31	38	17	29	21
Sum	110	88	117	58	102	90
**Improvement (%)**		**20.0**		**50.4**		**11.8**

We did not observe changes in appetite, depression, or sexual behavior in the assessed patients. A summary of the observed complications is shown in Table 4.

**Table 4:** Observed complications in patients receiving DBS.

	**Complication**	**Onset**	**Cause**	**Action taken**
Patient 1*	No complications	Not applicable	Not applicable	Not applicable
Patient2	No complications	Not applicable	Not applicable	Not applicable
Patient 3	IPG malfunction	~42 months after implantation	Patient non-compliance (battery damage due to discharge)	IPG reimplanted (non-rechargeable)
Patient 4	Inflammatory complications of posterior hypothalamus DBS system	3 months after implantation	Self-destructive behavior—scratching the scar (MSSA infection)	Explantation of posterior thalamus DBS system
Patient 5**	No complications	Not applicable	Not applicable	Not applicable
Patient 6	No complications	Not applicable	Not applicable	Not applicable

* *Patient 1, Inflammatory complication (MSSA infection) of GPI DBS system after ~14 months—system explanted*.

** *Patient 5, Posterior hypothalamus DBS system impedance increase—maximum stimulation current reduced to 1.2 mA*.

## Discussion

**Figure 4:**
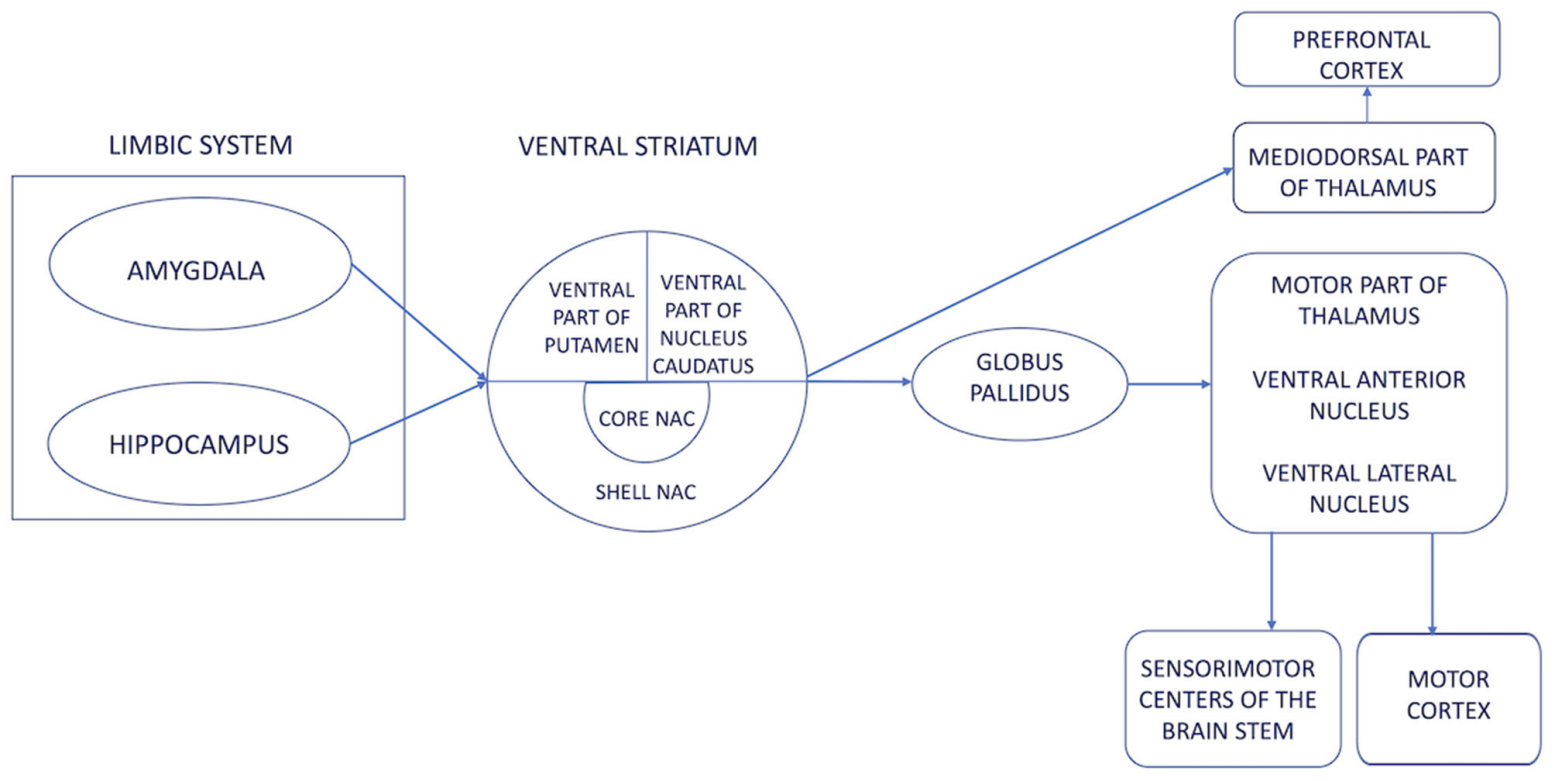
A diagram of the emotional-motor switch illustrating the connections of the limbic system with the motor cortex, i.e., the essence of aggressive and self-destructive behaviors. The ventral striatum is an area where the authors modulated pathological impulses coming from the limbic system to produce aggressive and self-destructive behavior.

The search for brain regions for DBS in patients with mood disorders, aggression, and OCD has a history at least as long as that of functional neurosurgery. While the nucleus accumbens septum has been used as a stimulation target in attempts to treat OCD and depression (21), this is the one of the first reports of encouraging results of nucleus accumbens DBS for aggressive and self-destructive behavior. One other case report of a 42-year old woman with autism similarly treated with DNS of the nucleus accumbens described positive results in terms of OCD symptoms, depression, and autism (22), and the nucleus accumbens has been observed to reduce aggression when performed for other indications (11, 23). Targeting the nucleus accumbens to treat aggressive and self-destructive disorders has both anatomical and neurophysiological foundations, since it acts as an emotional-motoric switch and mainly connects to the amygdala and hippocampus, i.e., the most important emotion-defining structures in the limbic system (24) (Figure 4).

We also observed situations in which patients reported significant deteriorations in their condition. While this did not happen often, in a few cases there was a device shutdown, battery discharge, or other dysfunction which resulted in a worsening condition noticeable by both patient and carers. These events, however, further demonstrated the efficacy of stimulation and made a placebo effect or patient desire to satisfy the treating physician less likely. We evaluated one patient with Tourette syndrome during one of these events and found that the patient's verbal aggression increased from 1 to 7 during device shutdown but decreased to 1 again when treatment resumed (pre-treatment MOAS of 11).

We previously reported the first of these cases as a case report (11) but stimulating the posterior medial hypothalamus based on the publications of Sano and Franzini (9, 10). While a rapid improvement was observed with this approach, within a few months the effects of stimulation wore off. Since the patient was also diagnosed with OCD, we chose the nucleus accumbens as a target and, while the improvement was slower, it has persisted for seven years. The second patient suffering from autism and epilepsy had to be treated in the same way, except with a stimulator implanted into the nucleus accumbens as a first step and, as a second step, stimulation of the posteromedial thalamus. While his clinical condition significantly improved after the first procedure, the second procedure did not further improve the patient's condition; indeed, before the first operation, the patient required direct coercive mechanical restraint (e.g., with belts and straps) 24 h a day, but this was not necessary after DBS. This lack of restraint, however, also meant that he was able to injure the implant site, and he developed inflammation around the implanted pulse generator (IPG) for the posteromedial hypothalamus, so this IPG was removed. Therefore, this patient was assessed after stimulation of the nuclei accumbens alone.

The clinical condition of patients presenting with Tourette's syndrome was significantly better than the other patients. They functioned independently and were able to complete the Buss-Perry aggression questionnaire on their own. The better outcomes obtained by patients with Tourette's syndrome may be due to their better preoperative condition.

Unfortunately, we cannot precisely define which part of nucleus accumbens was stimulated in our series. As noted by van den Munckhof et al. (25), DBS targeting the nucleus accumbens may actually stimulate the surrounding areas, which might be responsible for the therapeutic effect. Hence, stimulation of the surrounding ventral part of the anterior limb of the internal capsule may have contributed to the clinical response (as described in Figure 1).

This study was limited by the small group of patients who although had common symptoms and sequelae of self-destructive and aggressive behaviors had a variety of etiologies, so the population was heterogeneous. Furthermore, the evaluations were retrospective and administered at different times after surgery. Since the precise determination of stimulated anatomical structures at such a high current is not always possible and we used only MRI T1 and T2 images to determine the nucleus accumbens, the entire abdominal striatum may have been stimulated. Precise localization of the stimulated anatomy by tractography would be beneficial in any future, prospective study to accurately map anatomy to functional outcomes.

Although another recent case series described successful outcome by DBS of the posteromedial hypothalamus (26), this is the largest group of patients reported in the literature treated neurosurgically for aggression and self-destruction by DBS of the nucleus accumbens. A common characteristic of the posteromedial hypothalamus and nucleus accumbens is the multiple connections with the limbic system (amygdala and hippocampus) and the independent connections of both structures with the thalamus, motor cortex, and the brainstem. Based on our experience, blocking the hypothalamus provided a good but short-lived effect, while the action of high-intensity current from the deepest activated contacts of the electrode impacted the function of the entire ventral striatum to deliver a long-lasting effect. Perhaps the combination of both targets, as in case number one, or individual selection of the target on the basis of clinical, biochemical, and imaging studies will allow more effective target selection in the future. Further, the heterogeneous etiologies but similar outcomes may indicate that DBS of the nuclei accumbens treats a common mechanistic pathway and relieves symptoms regardless of etiology. This is important for patient recruitment and inclusion in future studies, because it is not uncommon for patients suffering from aggression and self-destructive disorders to have different, sometimes even contradictory diagnoses, so a common symptomatologic axis may be sufficient for qualification for surgical treatment. Bilateral stimulation of the nucleus accumbens may be an effective treatment for aggressive and destructive behaviors of multiple etiologies. Further prospective studies are warranted.

## Data Availability Statement

The original contributions generated for the study are included in the article/Supplementary Material, further inquiries can be directed to the corresponding author.

## Ethics Statement

The studies involving human participants were reviewed and approved by Military Medical Chamber Bioethics Commission. The patients/participants provided their written informed consent to participate in this study. Written informed consent was obtained from the individual(s) for the publication of any potentially identifiable images or data included in this article.

## Author Contributions

MH coordinated and performed the procedures. MR and MK performed the procedures. MK reported the study results, was responsible for the interpretation of the clinical data, supervised the study and proofread the article. MH, MR, JF, and MB contributed to the conception and design of the study. MK collected the data and performed the statistical analysis. MK and MH wrote the first draft of the manuscript. All authors contributed to the article and approved the submitted version.

## Conflict of Interest

The authors declare that the research was conducted in the absence of any commercial or financial relationships that could be construed as a potential conflict of interest.

## Publisher's Note

All claims expressed in this article are solely those of the authors and do not necessarily represent those of their affiliated organizations, or those of the publisher, the editors and the reviewers. Any product that may be evaluated in this article, or claim that may be made by its manufacturer, is not guaranteed or endorsed by the publisher.
