# Supplementary material for: Treating Aggression and Self-destructive Behaviors by Stimulating the Nucleus Accumbens: A Case Series
Source: Front Neurol. 2021 Oct 11;12:706166. doi: 10.3389/fneur.2021.706166 (PMC8542713; doi:10.3389/fneur.2021.706166)
Supplement: Supplementary file 1 [file Table_1.DOCX]

Supplementary Materials

**Individual trajectories and stimulation targets for each patient**

**Patient 1**

**
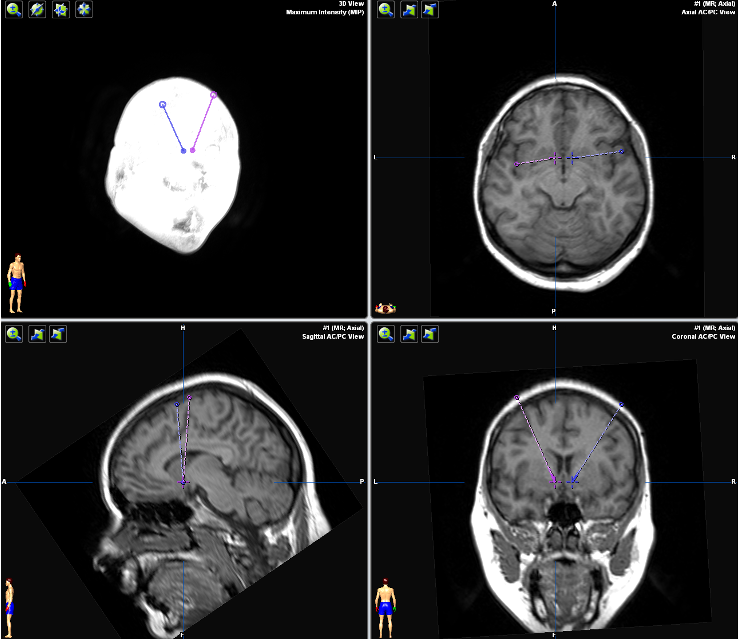
**

**Patient 2**

**
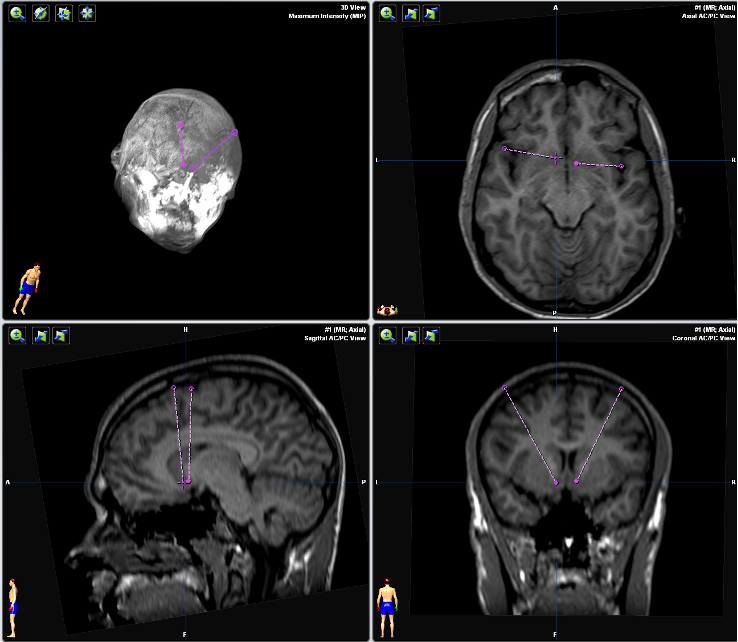
**

**Patient 3**

**
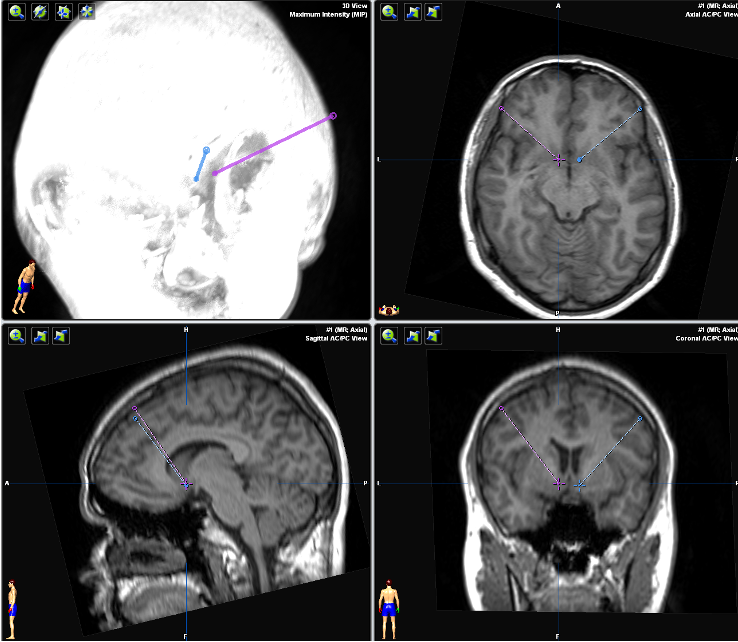
**

**Patient 4**

**
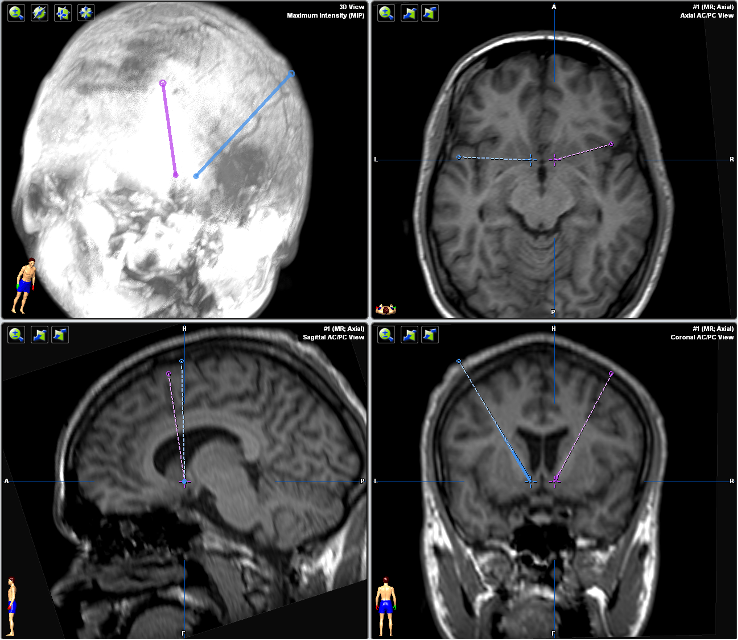
**

**Patient 5**

**
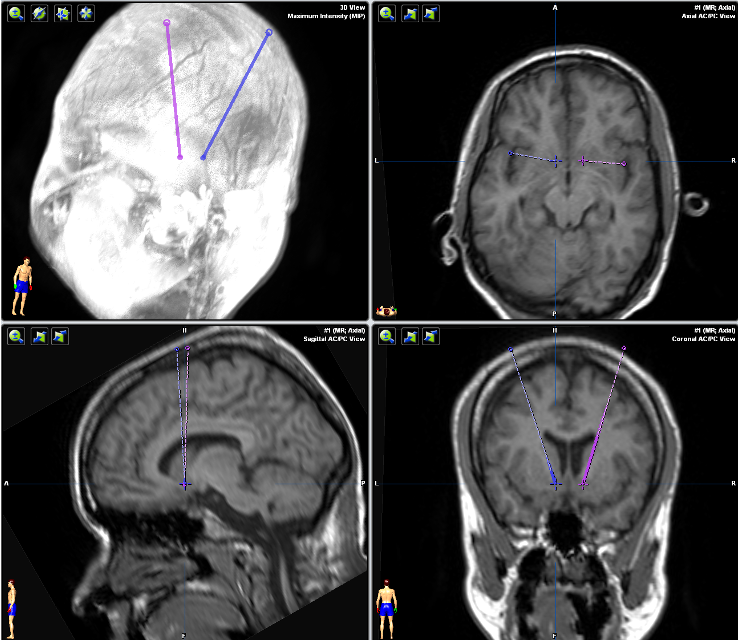
**

**Patient 6**

**
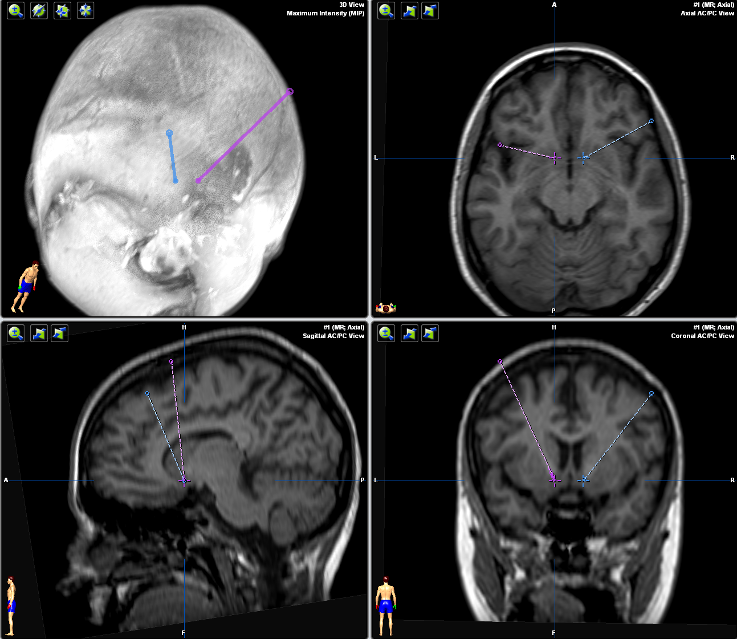
**
